# 

**Published:** 1909-10-15

**Authors:** 


CONTENTS

Event and Comment - - - - - - - - 497

A Consideration of the Casting Process, with Special Reference to Refractory Materials, - *By Marcus L. Ward, D.D.Sc.* 503

"How Dry I Am!" - - - *By George Zederbaum, D.D.S.* 522

The Making of Inlays by the Impression Method, *By F. T. Van Woert, M.D.S.* 528

The Extraction of Teeth under Nitrous Oxide Anesthesia, *By Alfred W. Hall, D.D.S.* 534

Obituary - - - - - - - - - - - - 539

Bibliography - - - - - - - - - - 540

Indents - - - - - - - - - - - - 544

Announcements - - - - - - - - - - 548

